# Optimisation of Epoxide Ring-Opening Reaction for the Synthesis of Bio-Polyol from Palm Oil Derivative Using Response Surface Methodology

**DOI:** 10.3390/molecules26030648

**Published:** 2021-01-27

**Authors:** Norsuhaili Kamairudin, Seng Soi Hoong, Luqman Chuah Abdullah, Hidayah Ariffin, Dayang Radiah Awang Biak

**Affiliations:** 1 Higher Education Centre of Excellence (HiCoE), Institute of Tropical Forestry and Forest Product, University Putra Malaysia, Serdang 43400, Selangor, Malaysia; suhailikamairudin@gmail.com (N.K.); hidayah@upm.edu.my (H.A.); 2 Malaysian Palm Oil Board, No. 6, Persiaran Institusi, Bandar Baru Bangi, Kajang 43000, Selangor, Malaysia; 3 Department of Chemical and Environmental Engineering, Faculty of Engineering, University Putra Malaysia, Serdang 43400, Selangor, Malaysia; dradiah@upm.edu.my; 4 Faculty of Biotechnology and Biomolecular Sciences, Universiti Putra Malaysia, Serdang 43400, Selangor, Malaysia; 5 Institute of Advanced Technology, Universiti Putra Malaysia, Serdang 43400, Selangor, Malaysia

**Keywords:** methyl oleate, epoxidation reaction, mechanism, central composite rotatable design (CCRD), hydroxyl value, nuclear magnetic resonance (NMR)

## Abstract

The development of bio-polyol from vegetable oil and its derivatives is gaining much interest from polyurethane industries and academia. In view of this, the availability of methyl oleate derived from palm oil, which is aimed at biodiesel production, provides an excellent feedstock to produce bio-polyol for polyurethane applications. In this recent study, response surface methodology (RSM) with a combination of central composite rotatable design (CCRD) was used to optimise the reaction parameters in order to obtain a maximised hydroxyl value (OHV). Three reaction parameters were selected, namely the mole ratio of epoxidised methyl oleate (EMO) to glycerol (1:5–1:10), the amount of catalyst loading (0.15–0.55%) and reaction temperature (90–150 °C) on a response variable as the hydroxyl value (OHV). The analysis of variance (ANOVA) indicated that the quadratic model was significant at 98% confidence level with (*p*-value > 0.0001) with an insignificant lack of fit and the regression coefficient (R^2^) was 0.9897. The optimum reaction conditions established by the predicted model were: 1:10 mole ratio of EMO to glycerol, 0.18% of catalyst and 120 °C reaction temperature, giving a hydroxyl value (OHV) of 306.190 mg KOH/g for the experimental value and 301.248 mg KOH/g for the predicted value. This result proves that the RSM model is capable of forecasting the relevant response. FTIR analysis was employed to monitor the changes of functional group for each synthesis and the confirmation of this finding was analysed by NMR analysis. The viscosity and average molecular weight (MW) were 513.48 mPa and 491 Da, respectively.

## 1. Introduction

Polyol is known to be a very important and beneficial material in pharmaceutical, food science and polymer chemistry [1,2,3]. Polyol is commonly used as raw materials in polymer chemistry in order to manufacture polyurethane products (coatings, elastomers and foam) by reacting with isocyanates [4,5,6]. Therefore, due to its versatility, polyurethane is extensively used in daily life and it has been predicted that the demand will expand from 14.2 million tons in 2011 to 22.2 million tons by 2020 [7]. However, the recent polyol industry has been heavily dependent on petroleum resources, which aggravates the depletion of inadequate natural resources. In view of the current emphasis on sustainability and global warming, the production of bio-based polymers from renewable resources has become of great importance [8]. 

Historically, vegetable oils such as soy bean oil [9], palm oil [10,11], canola oil [12] and jatropha oil [13] are the major renewable resources used for synthesising bio-polyol. For the best commercial plantation, palm oil has a yield potential of usually about 4 to 6 tons of oil per hectare per year. Approximately, 17.86 million tons of crude palm oil were produced in 2019 in Malaysia alone [14]. It has been predicted to increase to over 20 million tons in 2020. Therefore, palm oil and its derivatives are a great eco-friendly renewable resource for bio-based polyurethane materials due to their low cost and abundance of supply [10,15]. Palm oil consists of 53.4% saturated and 46.6% unsaturated fatty acids. The major fatty acids are palmitic acid, oleic acid and linoleic acid [10]. However, due to the absence of hydroxyl group in the structure of palm oil and its derivatives, they are unreactive under the condition of polyurethane chemistry. Therefore, they need to be functionalised with the introduction of the hydroxyl group in the molecular structure, so that they can be employed to produce polyurethane products. The introduction of the hydroxyl group can be undertaken at the position of alkene by various methods [16]. Example of methods employed are ozonolysis and hydrogenation [17], epoxidation followed by ring opening [18], thiol-ene coupling [19], and microbial conversion [20].

Methyl oleate (MO) is one type of fatty acid methyl ester (FAME), also recognised as biodiesel, derived from the transesterification of fats or vegetable oils such as palm oil, soybean oil and rapeseed oil. Due to the abundant availability of palm oil, this situation drives Malaysia towards the development of biodiesel technology and production. The current biodiesel production capacity in Malaysia is about 10.2 million tons [21]. However, the Malaysian biodiesel industry faces some challenges due to insignificant public support and escalating nontariff barriers, which indirectly influence the demand on for palm oil biodiesel [22]. Diversification of methyl oleate (MO) into other chemical products would assist to decrease the excess supply and capacity of the biodiesel industry. Thus, functionalisation of methyl oleate by introducing the hydroxyl functional group may develop new and useful bio-based products such as bio-polyol for use in the production of polyurethane products.

As reported by Hazmi et al., [13], the epoxidation of jatropha oil followed by a hydroxylation process using methanol, distilled water and formic acid as a catalyst generates polyol with a hydroxyl value of 179 mg KOH/g. The same chemical modification can be seen through the study by Tuan Ismail et al., [21], in which the hydroxyl value for the FAME polyol synthesised via epoxidation and ring opening process with ethylene glycol and boron trifluoride diethyl ether complex was about 189 mg KOH/g, which limits its use in some polyurethane applications such as rigid polyurethane foam. Based on extensive studies in the literature concerning the synthesis of bio-polyol through epoxidation followed by ring opening reaction, there has been no report on the optimisation of the reaction condition for this process as a function of the hydroxyl value (OHV). Therefore, the optimisation process for the hydroxyl value can be carried out using a competent method to evaluate the effect of the individual factors of each reaction condition on the overall effectiveness. One of the methods is Response Surface Methodology (RSM) that was first introduced by Box and Wilson in the 1950s. RSM is a mathematical technique that has been widely used in various areas of industry and academic research in order to optimise experimental conditions [23]. In the normal technique, one factor at a time is employed to investigate the effect of the operational parameters on the respective response. However, this RSM tool can be used to discover the interaction between independent variables (factors) and dependent variables (responses) involving statistical analysis and mathematical modelling with a minimal number of experiments. Thus, it gives an indication of the optimal condition of the process [24,25].

The aim of this present study is to develop the RSM model to optimise the hydroxyl value of bio-polyol from methyl oleate. To the best of the knowledge of the authors, there is no reported study regarding the optimisation of the reaction condition for the epoxide ring opening reaction using RSM, even though it is used as a statistical tool for the optimisation technique in other chemistry areas such as extraction of flavonoids [23] and waste water treatment [26]. 

## 2. Results and Discussions

### 2.1. Epoxidation of Methyl Oleate (EMO)

#### Reaction Mechanism of Epoxidation of Methyl Oleate (EMO)

**Scheme 1:**
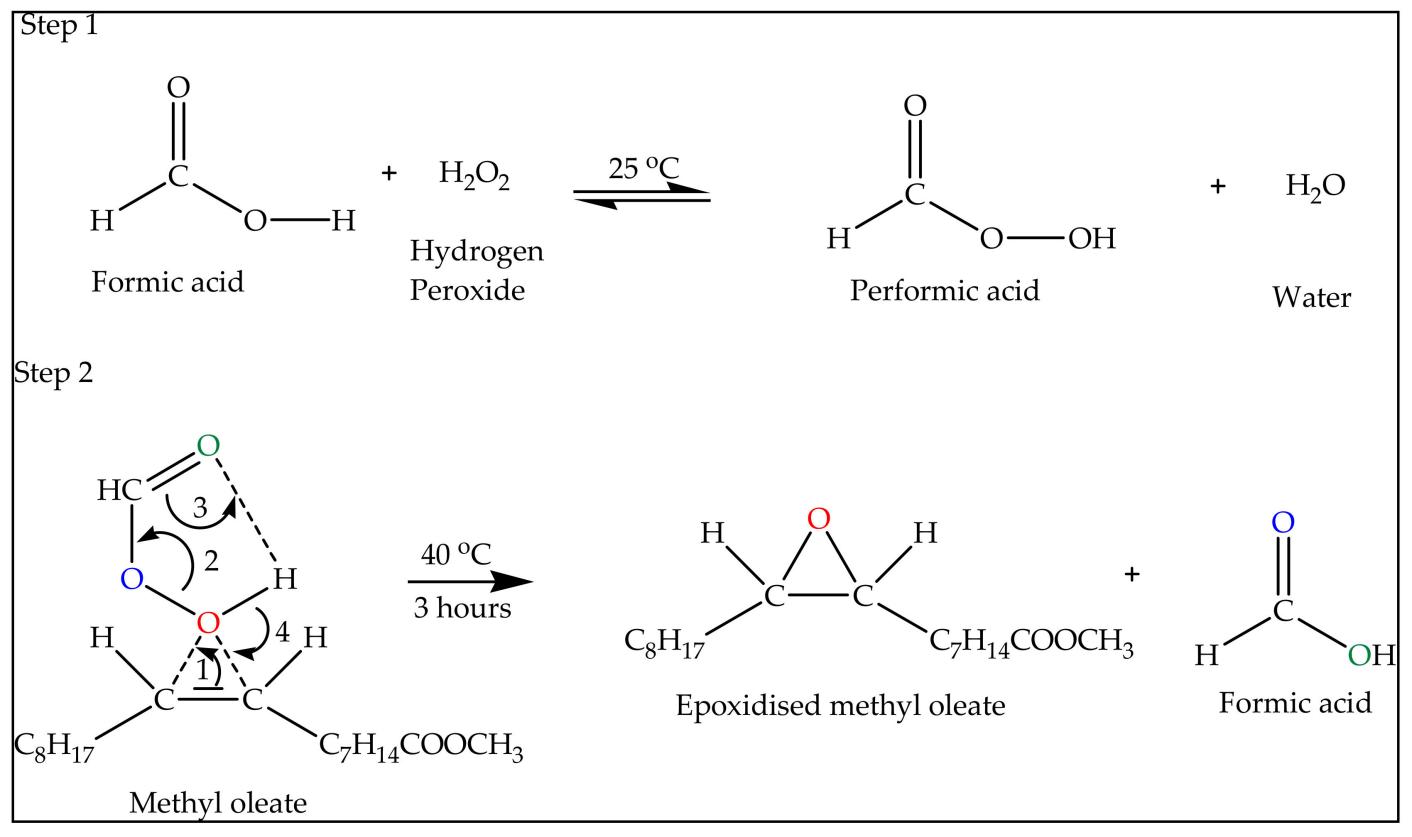
Reaction mechanism of epoxidation reaction for methyl oleate.

Scheme 1 illustrates the reaction mechanism of epoxidation of methyl oleate reaction. Step 1 shows that, after the addition of hydrogen peroxide, the performic acid was formed immediately. However, the performic acid was unstable and reactive. Thus, to prevent an extremely vigorous reaction, hydrogen peroxide was added dropwise. The oxirane ring (Step 2) was formed when the alkene group of methyl oleate reacted with performic acid, which yielded epoxidised methyl oleate. In order to get the highest oxirane conversion in the shortest time, excess hydrogen peroxide was needed to be used [27]. Therefore, in this present study, the mole ratio of EMO/formic acid/hydrogen peroxide was set at 1:1:2.5.

**Figure 1:**
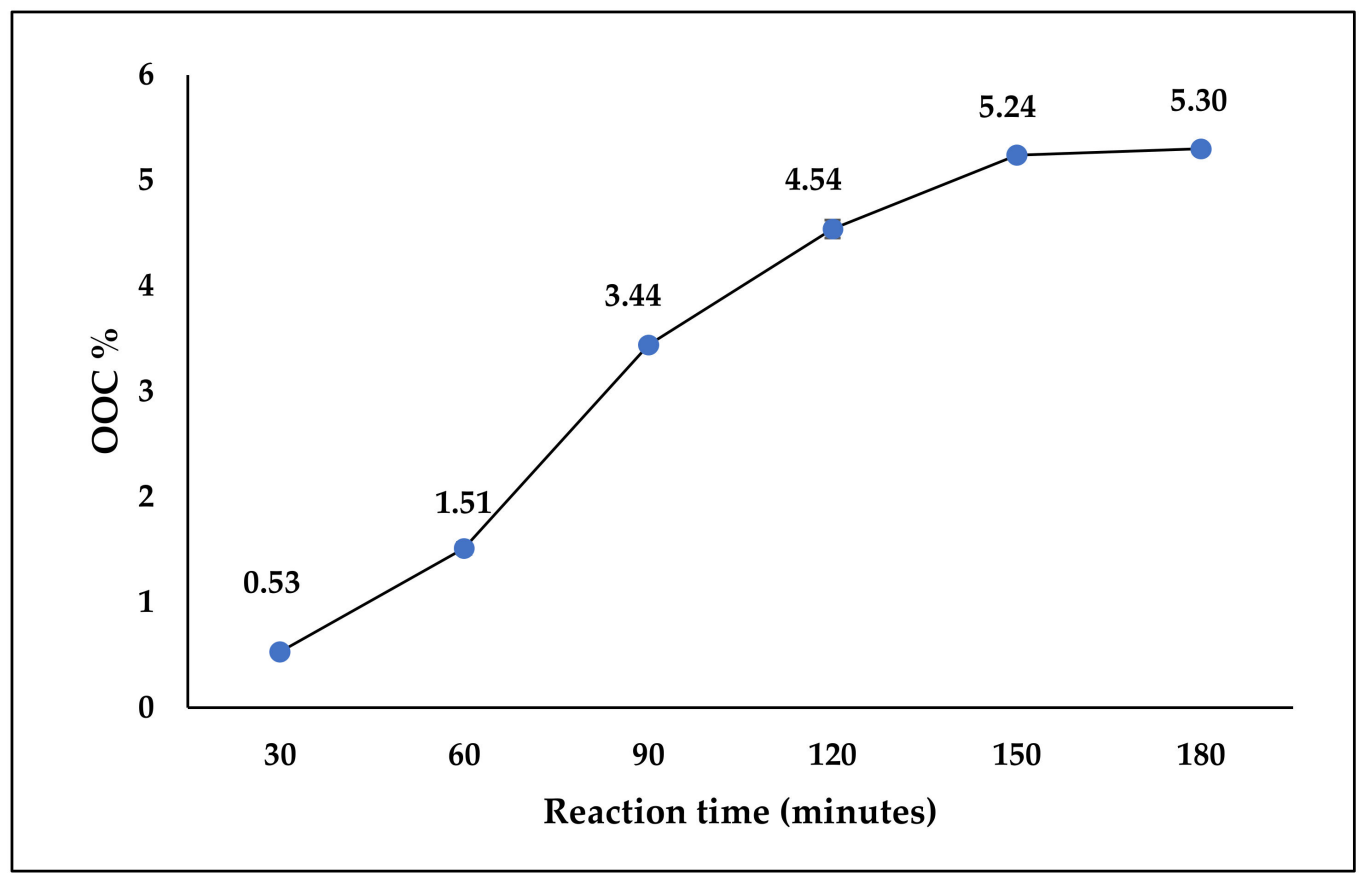
Oxirane oxygen profile for epoxidation reaction of methyl oleate.

Figure 1 shows the Oxirane Oxygen Content (OOC) profile for epoxidation reaction of methyl oleate (MO). This epoxidation reaction was completed within 3 h. The maximum OOC recorded in this reaction was 5.30%. However, after the washing and neutralisation processes, the final OOC recorded was 5.10%. This is attributable to the ring opening of some of the epoxide groups [21]. The final percentage of conversion was 93.38%. 

**Figure 2:**
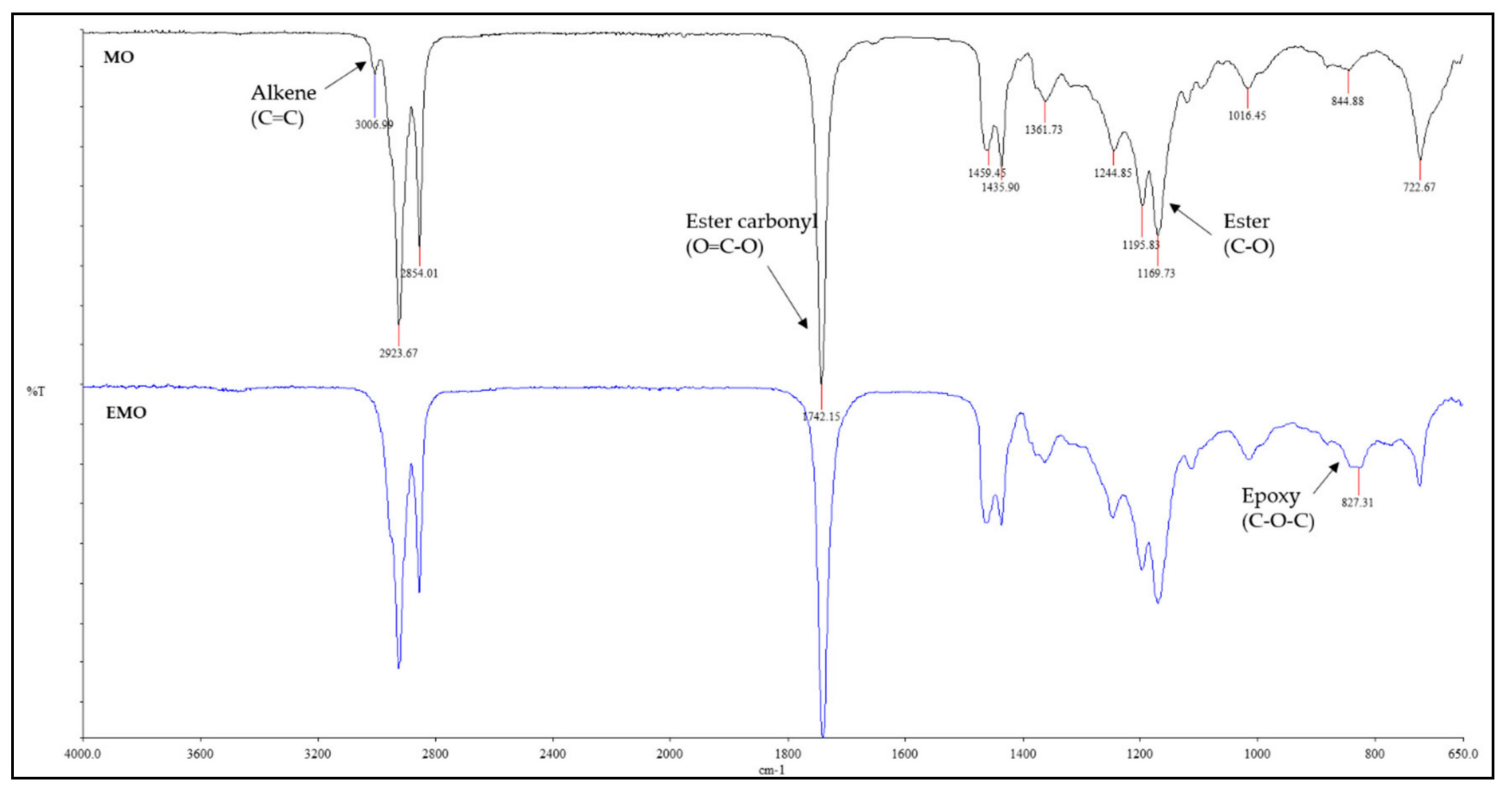
FTIR spectra of Methyl oleate (MO) and Epoxidised methyl oleate (EMO).

Figure 2 illustrates the comparative FTIR spectra for the MO and EMO. The chemical changes from MO to EMO can be clearly seen in the spectra. In the original state, the double bond (C=C) of the methyl oleate (MO) was detected as a small peak at 3007 cm^−1^. The peaks of the CH_2_ stretching vibration were presented at 2923 cm^−1^ and 2854 cm^−1^. The appearance of the characteristic ester carbonyl (C=O) was at 1742 cm^−1^, which confirmed the existence of the ester group in MO [21,28]. The peak of the ester functional group (C-O) appeared in the range of 1170 to 1240 cm^−1^ [21,29]. The peak at 722 cm^−1^ corresponded to the existence of the aliphatic chain. Upon the epoxidation reaction, the double bond of MO disappeared, and a new peak could be observed at 827 cm^−1^, which confirmed the formation of the epoxide group (C-O-C). According to Rayung et al., [30], the epoxide ring is usually detected at a bandwidth from 820–830 cm^−1^.

**Figure 3:**
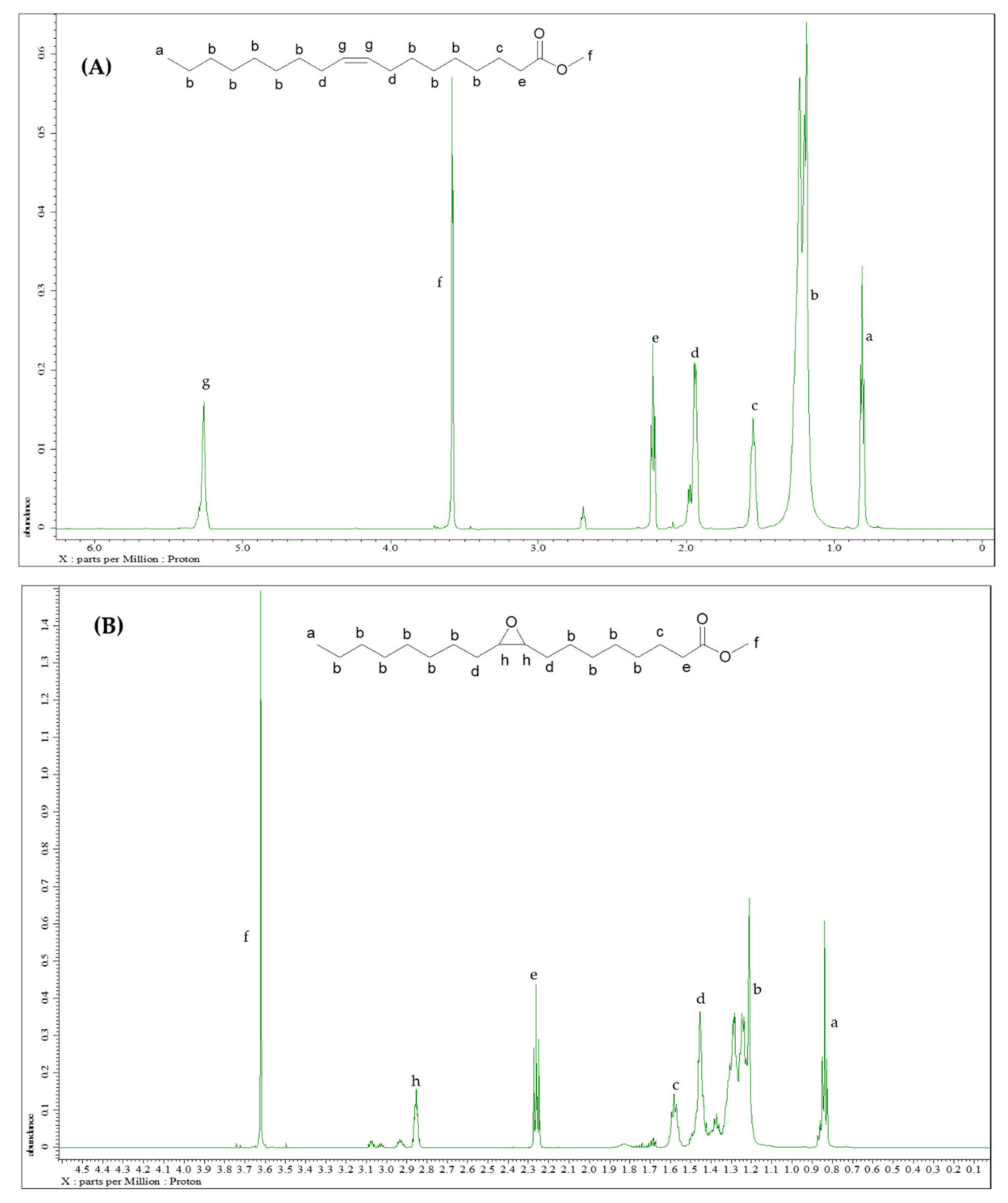
Proton NMR Spectra of (**A**) Methyl oleate and (**B**) Epoxidised methyl oleate (EMO).

Figure 3 illustrates the proton (^1^H) NMR spectra for MO and EMO. The molecular structure of MO and EMO were revealed using ^1^H-NMR analysis. In Figure 3A, the signal at 5.3 ppm (peak g) corresponds to the unsaturated double bond (C=C) that is present in MO. According to Sathai et al., [31], an unsaturated double bond signal can be seen at 5.2–5.41 ppm whereas signals for the long chain aliphatic (CH_2_) and terminal CH_3_ would appear at 1.2–1.4 (peak b) and 0.8–0.9 ppm (peak a), respectively. During the epoxidation process, the double bond (C=C) in MO was converted to an epoxide ring (C-O-C) in EMO. As shown in Figure 3B, a new peak of the epoxide ring (C-O-C) was observed at 2.88 ppm (peak h), which confirmed the formation of the epoxide group. This fact was also supported by the FTIR spectrum when the epoxide peak appeared at 827 cm^−1^. 

### 2.2. Ring Opening of Epoxidised Methyl Oleate with Glycerol

#### 2.2.1. Reaction Mechanism of Epoxide Ring-Opening by Glycerol

**Scheme 2:**
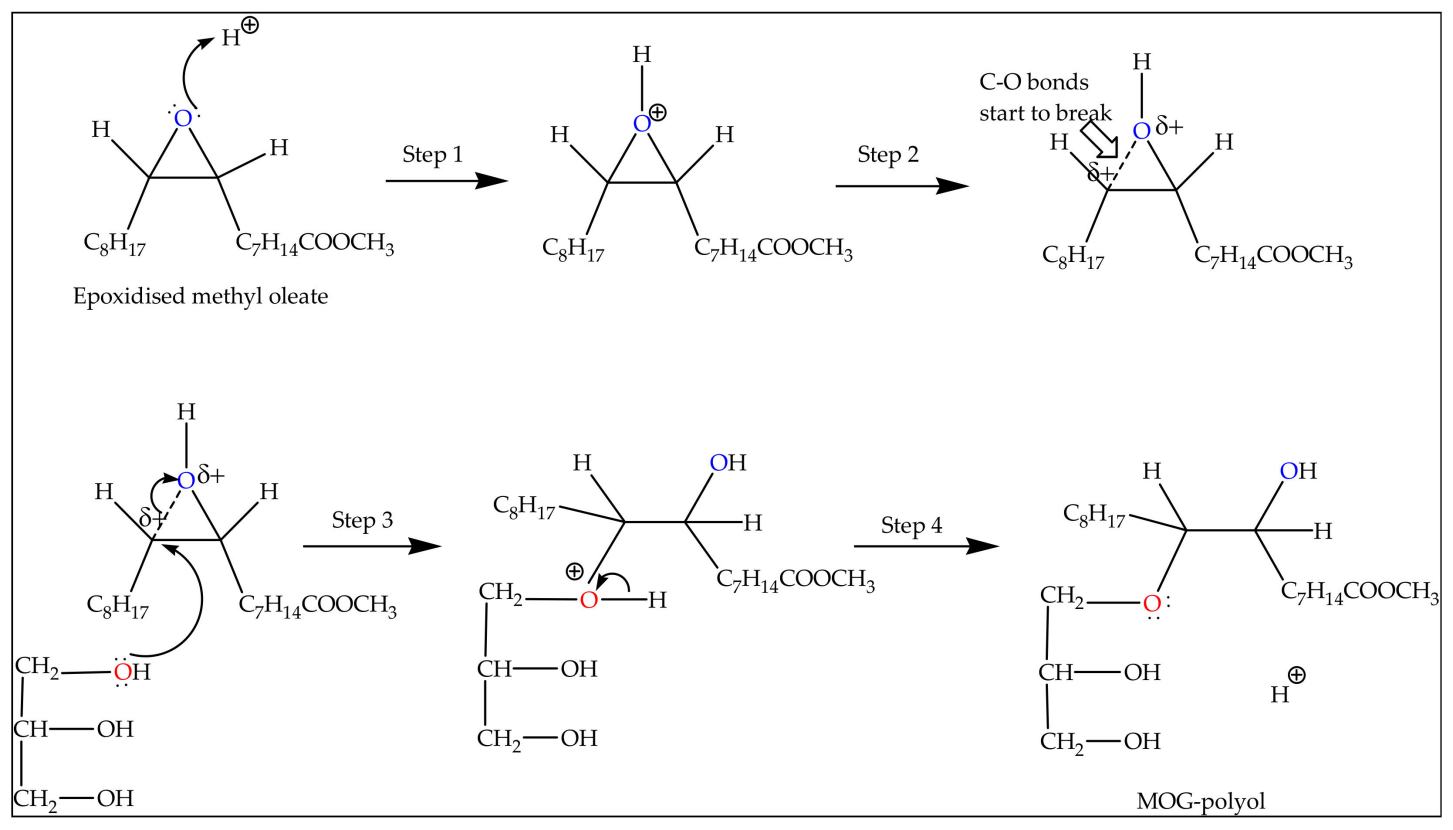
Reaction mechanism of epoxide ring-opening reaction with glycerol.

Scheme 2 illustrates the reaction mechanism of epoxide ring-opening by glycerol. In the first step, the oxygen atom of epoxide was protonated by a Lewis acid (BF_3_·Et_2_O) catalyst and generated a good leaving group (Step 1). After that, the carbon-oxygen bond began to break (Step 2) and positive charge started to build up on the more substituted carbon. The nucleophile (from glycerol) attacked the electrophile carbon (Step 3). Then, the oxygen was deprotonated (Step 4) to form the MOG-polyol product.

#### 2.2.2. Single Factor Experiment

**Figure 4:**
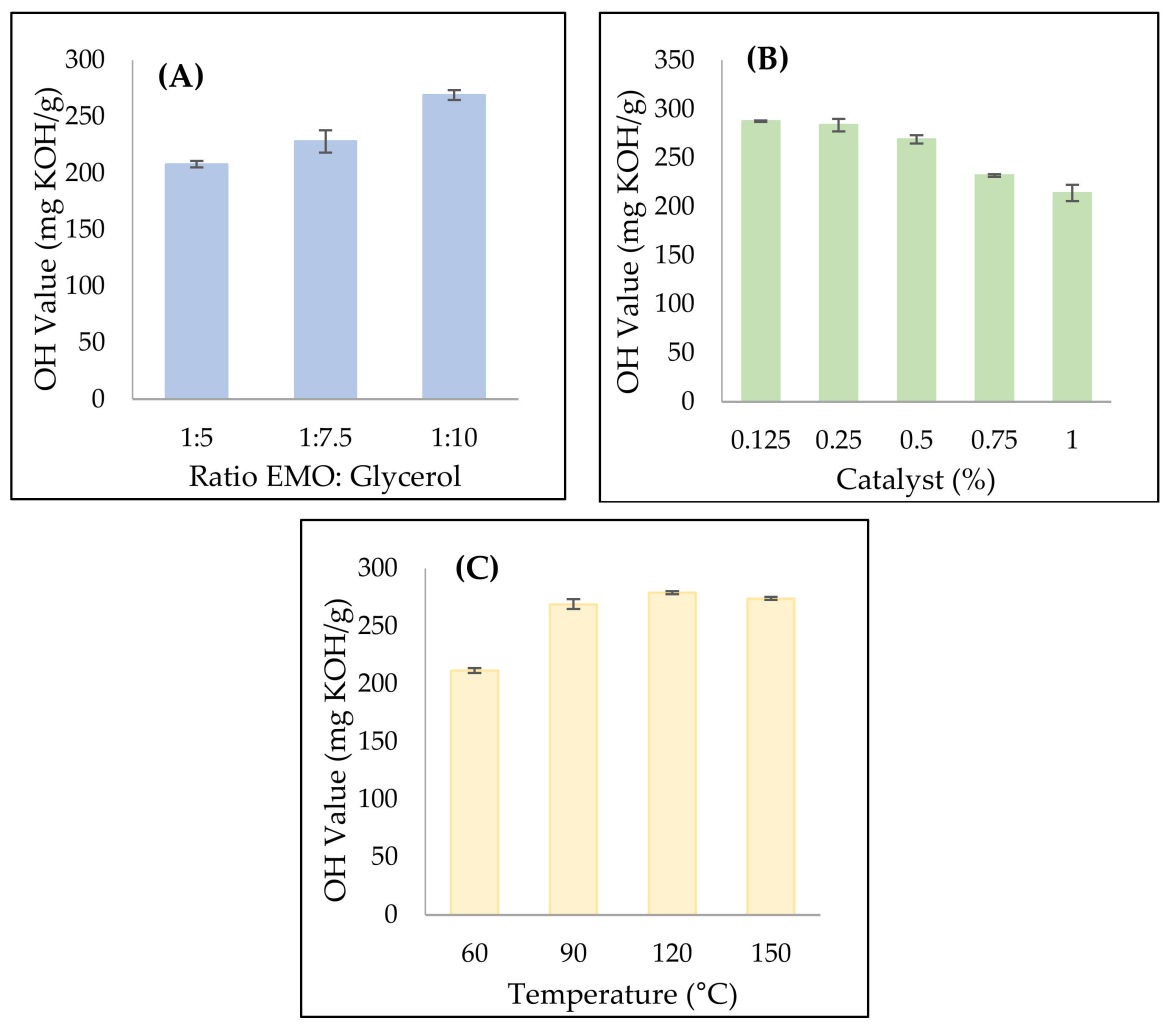
Effects of three factors on hydroxyl value. (**A**) Effect of mole ratio (EMO:Glycerol), (**B**) Effect of catalyst and (**C**) Effect of Temperature.

Figure 4 illustrates the results of the hydroxyl value (OHV) obtained at different mole ratios of (EMO:Glycerol), percentages of catalyst and reaction temperature.

##### Effect of Mole Ratio (EMO:Glycerol) on Hydroxyl Value (OHV)

In this experiment, the effect of mole ratio between EMO and glycerol on the hydroxyl value (OHV) of MOG-polyol was examined. Other reaction parameters were kept constant: catalyst loading 0.5%, reaction temperature at 90 °C. The mole ratio EMO:Glycerol was evaluated in the range between 1:5–1:10. The result obtained is shown in Figure 4A. The analysis result showed that the hydroxyl values of polyols increased with increasing mole ratio of EMO:Glycerol. The reason is that with a higher concentration of nucleophilic reactant (glycerol), the epoxide ring-opening reaction was favoured compared to the oligomerisation which leads an increase of OHV [32]. However, in this study, the mole ratio of EMO:Glycerol was not increased to more than 1:10 due to the high usage of glycerol as a reactant.

##### Effect of Catalyst on Hydroxyl Value (OHV)

In this part, the effect of the catalyst on the hydroxyl value (OHV) of MOG-polyol was studied. Other reaction parameters were kept constant, in which the mole ratio of EMO:Glycerol was 1:10, the reaction temperature was 90 °C and the catalyst loading was varied in the range of 0.125–1.0%. The result obtained is shown in Figure 4B. The data showed that the hydroxyl values of the polyols increased with decreasing amount of catalyst. Specifically, the hydroxyl value (OHV) increased from 208 mg KOH/g to 287 mg KOH/g as the catalyst decreased from 1.0–0.125%.

##### Effect of Temperature on Hydroxyl Value (OHV)

In this experiment, the effect of reaction temperature on the hydroxyl value (OHV) was explored, while the other reaction parameters were kept constant (mole ratio of EMO:Glycerol at 1:10, catalyst loading = 0.5%). The temperature was varied in the range of 60–150 °C. The result obtained is shown in Figure 4C. The obtained result showed that the hydroxyl values (OHV) of the polyols increased with increasing reaction temperature until 120 °C, beyond which the hydroxyl value (OHV) showed a slight reduction.

#### 2.2.3. Optimisation Reaction Conditions by Response Surface Methodology

##### Fitting the Model and Analysis of Variance (ANOVA)

Response surface modelling was applied to find the optimum reaction conditions for independent variables in order to obtain the maximum OHV for MOG-polyol. The experiments were programmed as per the CCRD layout using (Design Expert Software version 7.0, Stat-Ease, Minneapolis, MN, USA) containing 20 experiments with different combinations of independent variables. The design matrix of the actual and predicted OHV is shown in Table 1. Each experimental run was conducted in triplicate in order to obtain consistent results. The coefficients regressions were calculated based on CCRD and the results of the experiments. The second-order polynomial equation was established to obtain the hydroxyl value (OHV) as in Equation (1):

Y (Hydroxyl value) = + 280.83 + 16.43A − 18.15B + 2.05C + 7.08AB − 4.0AC − 0.81BC − 0.11A^2^ − 6.53B^2^ − 2.18C^2^ (1)

where A is the symbol of the mole ratio of EMO:Glycerol, B is the symbol of the catalyst, C is the symbol of the temperature and Y is the response (Hydroxyl value). A positive sign in the equation showed a synergistic effect. A negative sign indicated as an antagonistic effect [33,34]. 

Sometimes, a mathematical model developed after a fitting function to the data, may not satisfactorily express the experimental domain studied [33]. Therefore, a more reliable way to investigate the adequacy of a model and goodness fit for the response is by employing the analysis of variance (ANOVA) [35]. ANOVA analysis is also needed in order to discover whether the second-order polynomial model is significant or not. 

The quadratic polynomial model is illustrated in Table 2. The *p*-value (<0.0001) indicated that the developed model was significant and adequate to demonstrate the actual interaction between the significant variables and the response (hydroxyl value) [36]. The lack of fit (*F*-value = 4.18) showed insignificant data, which was comparative to the pure error of the experiments, which indicated that the quadratic model data were convenient to depict the whole experimental data. Noise in the experiment contributed to the large value of lack of fit [37]. The *p*-value was also used to distinguish the significance of every coefficient. A small *p*-value and large *F*-value would mean that the independent variables have a large impact and would be significant for represented response [38]. In addition, when *p*-value has a small value, the corresponding coefficient will be more significant. Table 2 illustrates that the coefficients of A, B, C, AB, AC, B^2^ and C^2^ were identified as significant in model terms due to the *p*-value being below 0.05. From the model, catalyst (B) (*F*-value = 453.02; *p*-value < 0.0001) was the most significant variable followed by the mole ratio (A) and temperature (C). However, from this model, BC and A^2^ did not show any significant effect, thus indicating that there is no mutual interaction between catalyst and temperature (BC) and mole ratio (EMO:Glycerol), (A) multiplied by itself. Although these model terms were insignificant, they were kept in the model equation in order to support the hierarchy. As the model design could accurately predict up to a 2% residual standard error (RSE) according to the model validation that meant the insignificant terms still contributed to the model. Ordinarily, insignificant terms are not eliminated from the equation, but may be retained depending on their suitability in the model.

The coefficient of determination (R^2^) can be a useful tool to make sure of the accuracy of the quadratic polynomial model as shown in Table 3. A high coefficient determination (R^2^ = 0.9897) was presented by the polynomial model, which suggested that 98% of the variation in the hydroxyl value (OHV) response could be described by the independent variables and only 2% of the variation could not be described by the model. Additionally, the predicted R^2^ (0.9296) was also in reasonable agreement with the adjusted R^2^ (0.9804), with a difference of less than 0.2 [39]. Therefore, the relationship between the predicted and experimental values of hydroxyl value (OHV) response was proven to have a high R^2^-value, which suggested that the experimental values fitted well with the predicted value [40]. 

In addition, the adequate precision demonstrated that a signal-to-noise ratio of greater than 4 is desirable [41]. The model showed that the result of an adequate precision ratio of hydroxyl value (OHV) response was 37.588, which indicated that the model could be employed to navigate the design space. For the model to be adequate, the value of the coefficient of variation (CV) result needed to be less than 10%. Therefore, as the study indicated a very low value (1.15%) for the CV, this showed that the experimental value had very good reliability and high precision [42].

**Figure 5:**
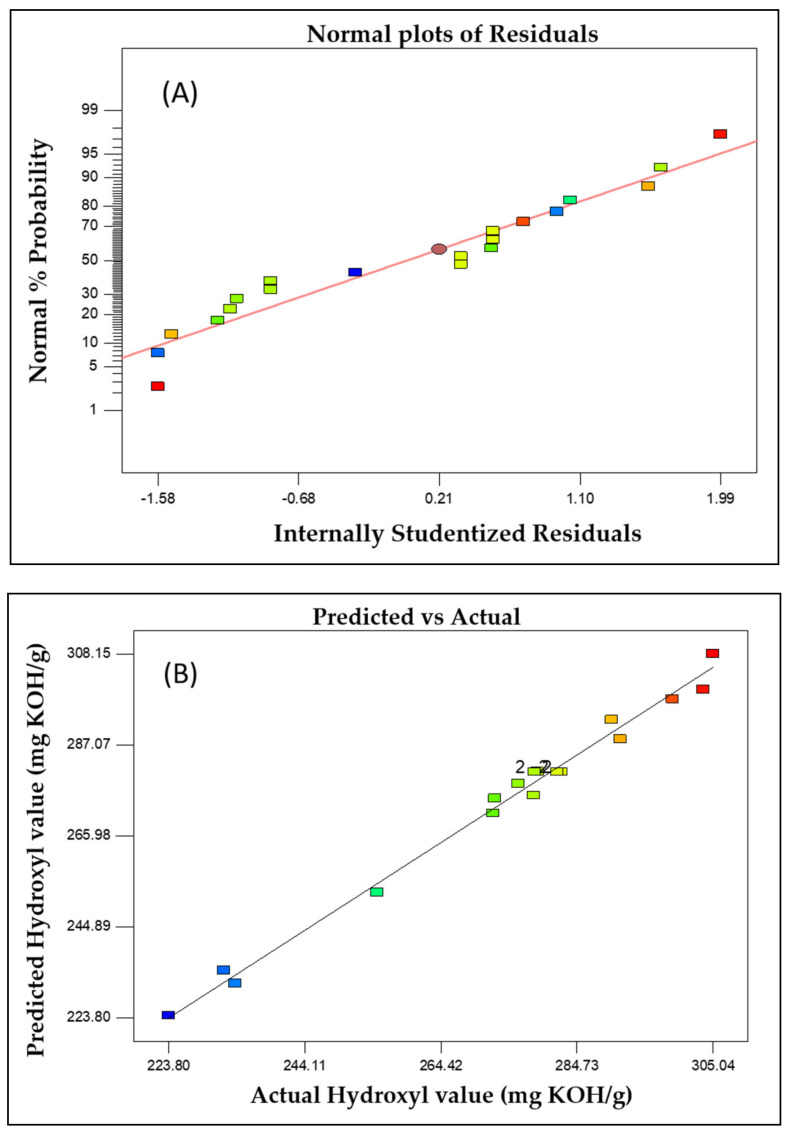
(**A**) The residual plot of experimental runs from central composite rotatable design (CCRD) and (**B**) scatter plot of predicted hydroxyl value (mg KOH/g) versus actual hydroxyl value (mg KOH/g).

The residual plot is a crucial detection method to discover the systematic departures from the assumption employed in the regression equations [43]. As shown in Figure 5A, it confirmed the independence of residuals and the assumption of a normal distribution, as the data points on this plot were close to a straight line. Figure 5B illustrates the correlation between the experimental and predicted values for the hydroxyl value (OHV) response. The actual values represented the measured experimental response data for a specific experimental run, while the predicted values were the prediction results generated by the model. As shown in Figure 5B, the actual values were correlated to the predicted values with an R^2^ of 0.9897. Therefore, this model predicted accurately to determine the correlation between the independent variables and the hydroxyl value (OHV).

##### Mutual Effects of Process Parameters

**Figure 6:**
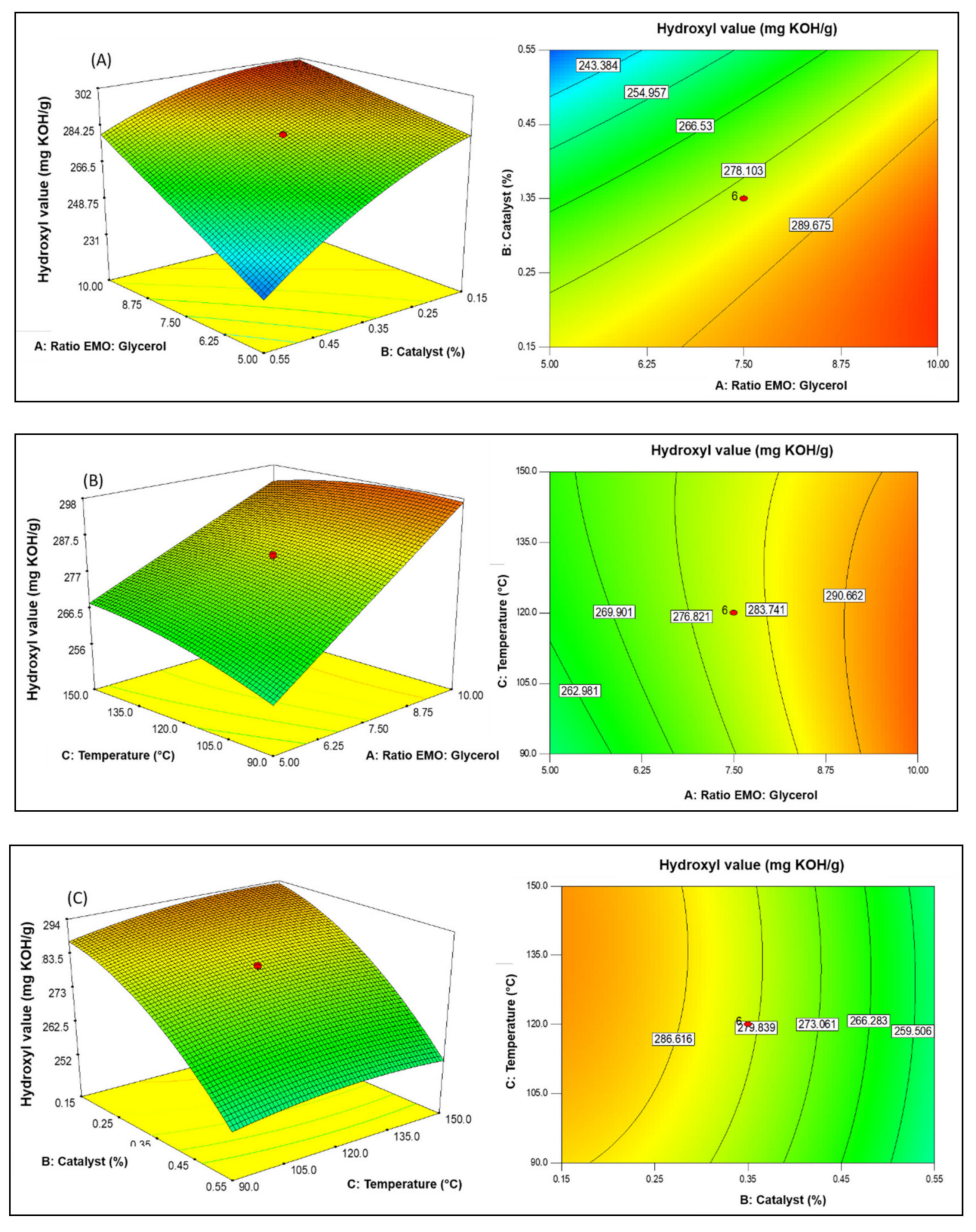
Response surface plots: (**A**) mole ratio (EMO:Glycerol) versus catalyst (%), (**B**) temperature (°C) versus Mole ratio (EMO:Glycerol) and (**C**) catalyst (%) versus temperature (°C).

The correlation between the independent variables provided significant impact on the hydroxyl value (OHV) response as shown in Equation (1). Instead of investigating one variable individually, a study about the correlation among variables could be done to determine their significance for more sophisticated optimisation. According to Mangili et al., [44], 2D (contour plot) and 3D (three dimensional graphs) of RSM model surfaces were illustrated as graphical presentation of the regression equation in order to have a better understanding of the interaction effect between two variables at a time within the range considered. Each 2D and 3D curve in Figure 6 depicts an infinite number of combinations of two significant variables while the other variables were kept at a fixed position at central level [41].

Figure 6A represents the interaction between the mole ratio of EMO to glycerol (A) and catalyst (B) on the hydroxyl value (OHV) response at a fixed temperature (C) of 120 °C. The OHV was positively correlated to the mole ratio (A), in which the OHV increased with a higher mole ratio. This was due to the high concentration of hydroxyl group of glycerol at a higher mole ratio. However, when the reaction was conducted with a higher loading of catalyst (B), the results showed lower OHV values. This may be due to the higher catalyst loading that promoted oligomerisation between the fatty ester chains to take place, which contributed to the lower hydroxyl values observed [45]. 

Figure 6B illustrates the relationship between the mole ratio (A) and temperature (C) as a function of the hydroxyl value (OHV) response at a fixed catalyst (B) of 0.35%. When the mole ratio of EMO:Glycerol was increased from 1:5 to 1:10, it caused the hydroxyl value to increase correspondingly. The same effect was observed in Figure 6A. The OHV increased with higher reaction temperature up to 120 °C, which was believed to be the optimum temperature. This might be due to deactivation of the catalyst (BF_3_·Et_2_O) because its boiling point is at 130 °C. Therefore, the catalyst was inefficient at the temperature above 130 °C.

Figure 6C illustrates the 2D and 3D plots of the correlation between catalyst (B) and the reaction temperature (C) on the OHV response at a fixed mole ratio (A) of (1:7.5). It was observed that the hydroxyl value (OHV) increased with decreasing catalyst loading such as when seen in the results shown in Figure 6A. For reactions conducted at temperatures in the range of 120–125 °C, the hydroxyl value (OHV) obtained ranged from 259–286 mg KOH/g.

### 2.3. Verification of the Model

A verification test was carried out to evaluate the validity of the model. In this part, three sets of experiment were produced from the Design-Expert Software (version 7.0, State Ease Inc., Minneapolis, MN, USA), in order to make a comparison between the predicted values and the experimental values. The maximum hydroxyl value (OHV) response was prescribed as a main target, while the other variables remained in the range studied (Table 4). Table 5 illustrates the actual and predicted responses from the optimum combination of the independent variables. For all experiments, the results showed a low residual standard error (RSE), which was less than 2%, thus indicating that the model was valid and could predict the OHV response up to 98%. RSE was calculated based on Equation (2). From Table 5, the highest OHV was obtained from Experiment 3. Thus, for this reason, Experiment 3 was chosen as the optimum reaction condition [46]. Therefore, based on CCRD-RSM, an optimised reaction condition with (1:10) mole ratio of (EMO:Glycerol), (0.18%) of catalyst and temperature of 120 °C was suggested, which would produce a polyol with hydroxyl value (OHV) of 306 mg KOH/g. Table 6 shows the optimised reaction conditions for the OHV response of the MOG-polyol with desirability about 0.976. A better-generated solution is given when the desirability is close to 1. Therefore, the optimum reaction condition for the maximum hydroxyl value (OHV) was successfully generated by the CCRD-RSM model.

(2) $$Residual\;Standard\;Error\;\left(RSE\;\%\right)=\;\frac{Experimental\;value-Predicted\;value}{Predicted\;value}\;\times 100\%$$

### 2.4. Evaluation of the Importance of the Variables on the Hydroxyl Value (OHV) of Polyol

**Figure 7:**
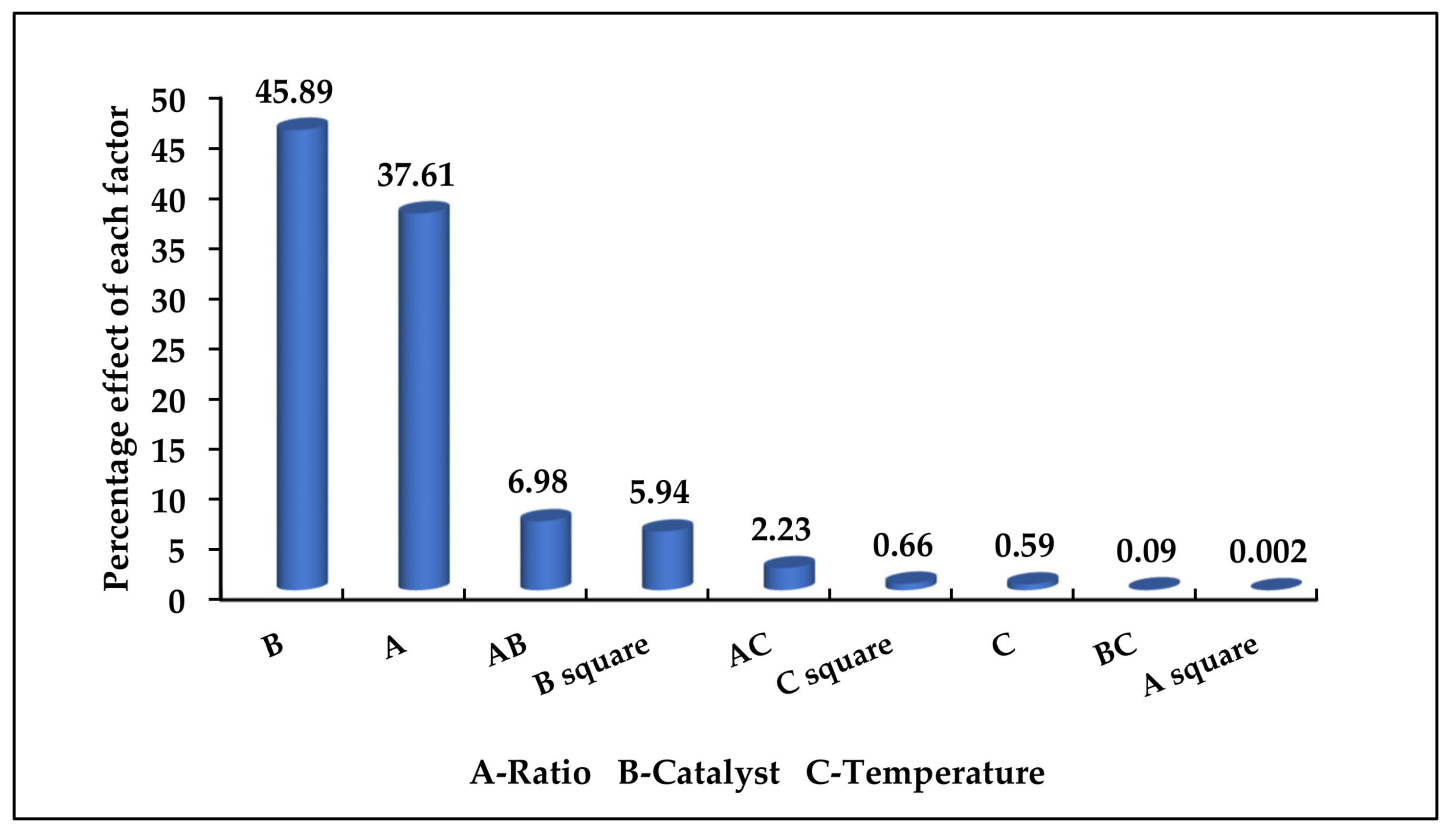
Pareto plot analysis describes the effects of each variables in percentage (%).

The important influence of efficient variables on the hydroxyl value was demonstrated by Pareto analysis as shown in Figure 7. The Pareto analysis expresses the influence of each variable, in percentages, on the response in relation to the coefficient of the coded variable in Equation (3) [33,47]. The pareto analysis showed that variable B (catalyst) gave the most important influence on the response (hydroxyl value) at 45.89% followed by variable A (mole ratio of EMO:Glycerol) at 37.61% and the interaction between A and B at 6.98%.

(3) $$P_{i}=\left(\frac{b_{i}^{2}}{\sum b_{i}^{2}}\right)\times 100\;\left(i\neq 0\right)$$

### 2.5. Characterisation of Optimised MOG-Polyol

**Figure 8:**
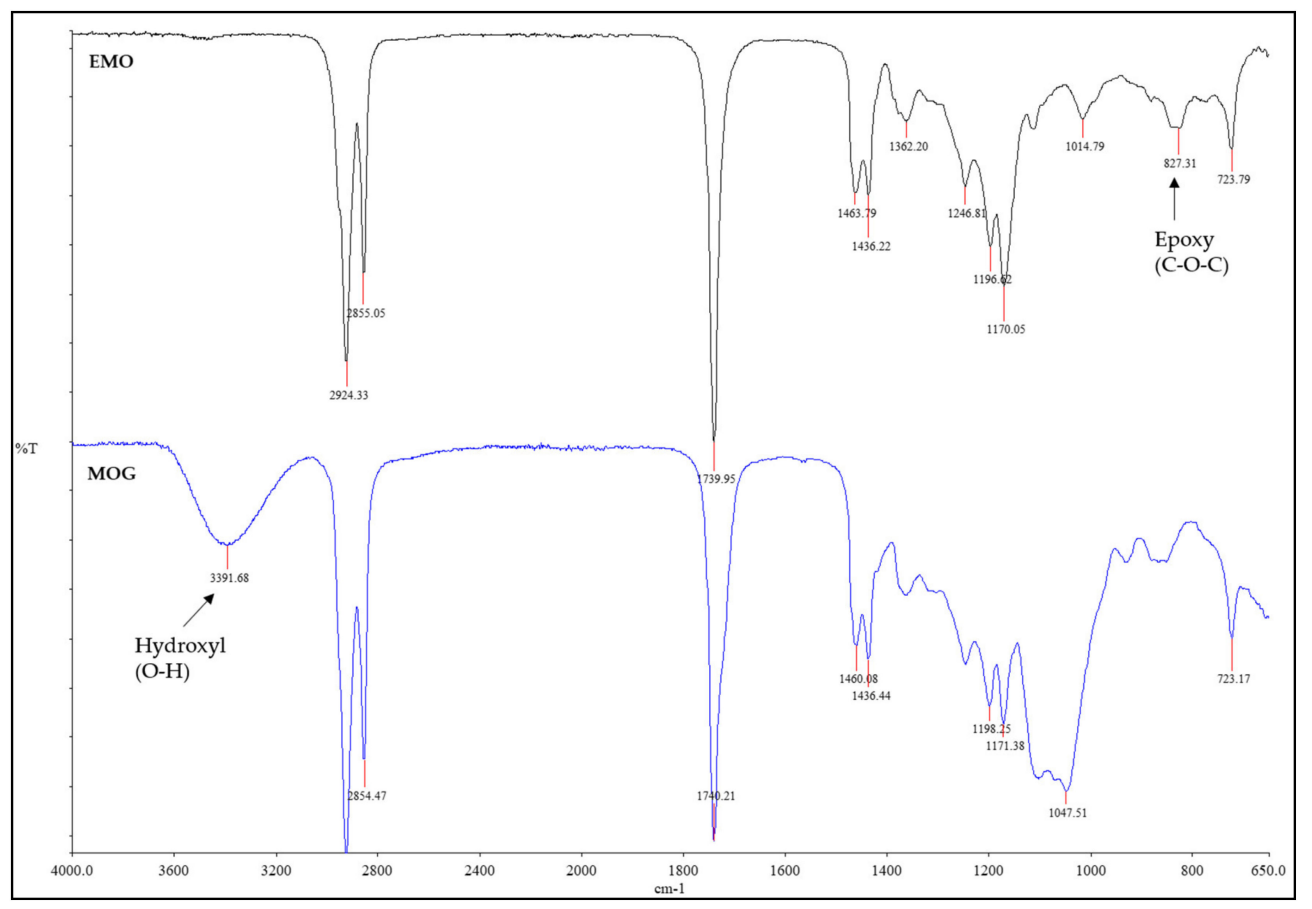
FTIR spectra for epoxidised methyl oleate (EMO) and MOG-polyol.

As shown in Figure 8, the disappearance of the epoxy group (C-O-C) vibration stretching at 827 cm^−1^ [47] for the MOG-polyol spectrum supported the fact that epoxide ring-opening reaction with glycerol had occurred. The reaction generated the hydroxyl group that was detected as a broad O-H peak at 3391 cm^−1^ that can be seen clearly in the FTIR spectrum [48]. The presence of this broad peak in this spectrum confirmed that the OH functional group had been added onto the epoxidised methyl oleate structure that originated from the glycerol molecule and the epoxide group.

**Figure 9:**
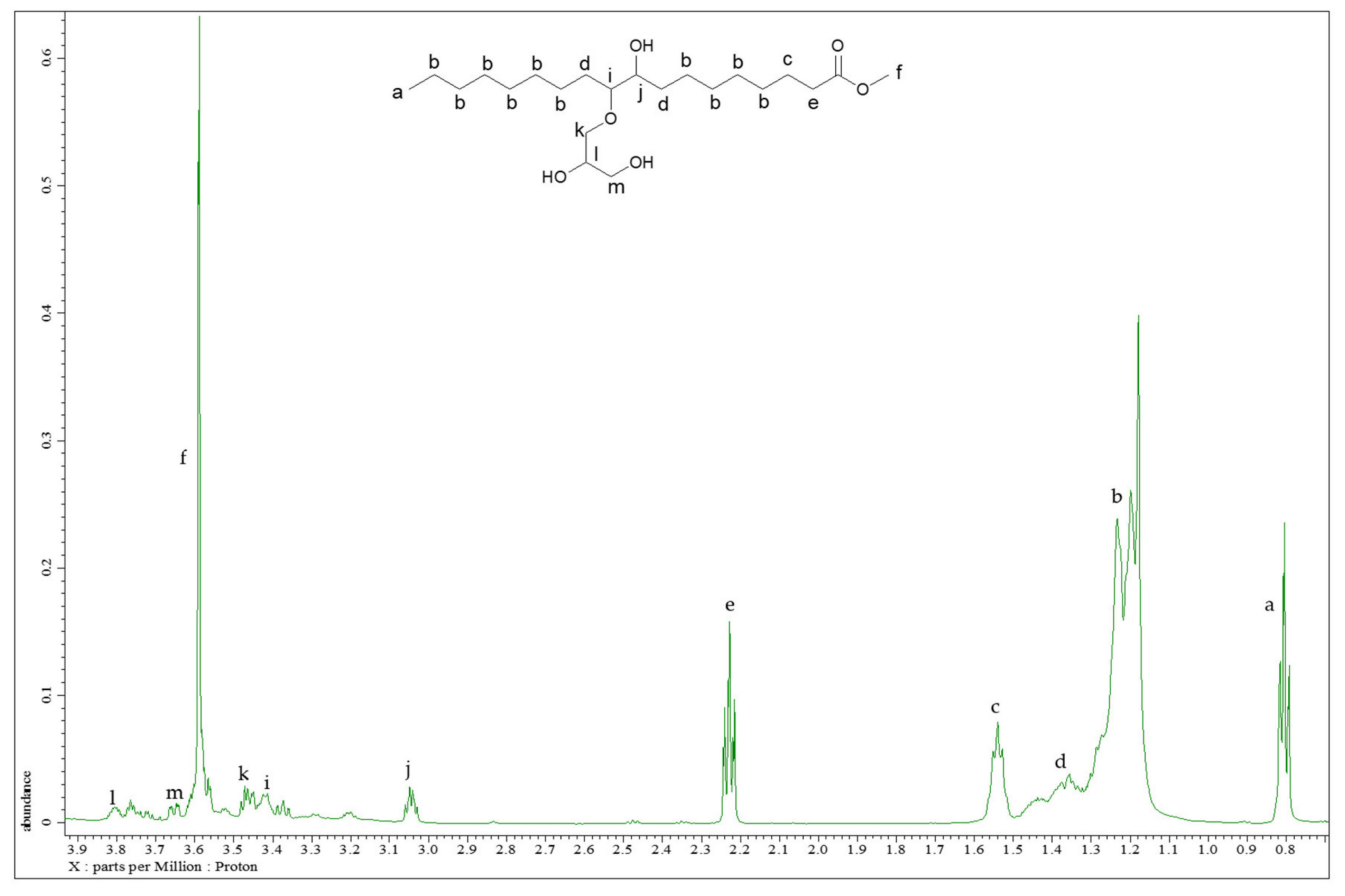
Proton NMR Spectra of MOG-polyol.

Figure 9 presents the ^1^H-NMR spectrum of the MOG-polyol, which confirmed that the peak associated with the proton of the epoxide group (C-O-C) at 2.88 ppm was not observed. This observation indicated that the epoxide ring-opening reaction with glycerol had occurred. Upon occurrence of the ring-opening reaction, new proton peaks were detected at 3.03–3.06 ppm, 3.41–3.48 ppm and 3.64–3.81 ppm, showing the presence of the OH groups in the MOG-polyol. This is supported by the appearance of a broad peak at 3319 cm^−1^ in the FTIR spectrum, which proved the presence of OH groups in the polyol.

The iodine value of the EMO (Table 7), which quantifies the alkene group, was significantly low in comparison with the MO due to epoxidation of alkene to the epoxide (C-O-C) group. As shown in Scheme 2, MOG-polyol contains both the primary and secondary OH group in its molecular structure, leading to a higher molecular weight as compared to EMO and MO (Table 7). Additionally, the hydroxyl groups of MOG-polyol could be quantified by wet chemistry analysis and were about 300 mg KOH/g. This result is in agreement with the finding by Prociak et al., [49]. The viscosity of the MOG-polyol was higher compared to EMO and MO due to the higher hydroxyl value (OHV) and molecular weight (MW) of the polyol [50]. 

## 3. Materials and Methods

### 3.1. Materials

Methyl oleate from palm oil with purity 98% and iodine number 87g I_2_/100g was purchased from Carotino Sdn Bhd (Pasir Gudang, Johor, Malaysia). Hydrogen peroxide (H_2_O_2_) (50% concentration in H_2_O), formic acid (94% concentration) and glycerol (99.5%) were purchased from (R&M Chemicals, Cennai, India). Boron trifluoride diethyl ether complex (BF_3_·Et_2_O) (50% purity) was purchased from Merck Sdn Bhd (Petaling Jaya, Selangor, Malaysia). Sodium chloride (NaCl), sodium carbonate (Na_2_CO_3_) and sodium hydrogen carbonate (NaHCO_3_) were purchased from Chem AR (Systerm Chemicals, Shah Alam, Malaysia), respectively. All purchased chemicals were used as received without further purification unless otherwise noted.

#### FTIR and NMR Data of Methyl Oleate

υ_max_/cm^−1^ 3007 (C=C), 2924, 2854 (CH_2_), 1742 (C=O), 1459 (CH_2_), 1436 (CH_3_), 1170 (C-O), 723 (CH_2_); ^1^H-NMR (600 MHz, CDCl_3_): δ_H_ = 5.30–5.24 (2H, m, CH_2_CH=CHCH_2_), 3.58 (3H, s, OCH_3_), 2.27 (2H, t, O=CCH_2_CH_2_), 2.00–1.94 (4H, m, CH_2_CH=CHCH_2_), 1.58–1.52 (2H, m, O=CCH_2_CH_2_CH_2_), 1.23–1.19 (20H, m, CH_2_CH_2_CH_2_), 0.81 (3H, t, CH_2_CH_3_); ^13^C-NMR (150 MHz CDCl_3_): δ_C_ = 174.1 (CH_3_OC=O), 130.1–129.6 (CH_2_CH=CHCH_2_), 51.3 (CH_3_O), 34.1 (O=CCH_2_CH_2_), 31.9–29.1 (CH_2_CH_2_CH_2_), 27.2–27.1 (CH_2_CH=CHCH_2_), 24.9 (O=CCH_2_CH_2_CH_2_), 22.6 (CH_2_CH_3_), 13.9 (CH_2_CH_3_).

### 3.2. Epoxidation of Methyl Oleate 

The epoxidation of methyl oleate was performed in a 5-L water-jacketed glass reactor, equipped with a mechanical agitator, a thermometer couple, a dropping funnel and reflux condenser. Methyl oleate (1000 g) was heated to 40 °C. Performic acid, a premixed reagent of 50 wt% hydrogen peroxide and formic acid of the desired ratio was slowly added dropwise to the preheated methyl oleate. The mole ratio of the methyl oleate/formic acid/hydrogen peroxide was 1:1:2.5. Oxirane oxygen content (OOC) analysis was used to monitor the progress of the epoxidation reaction. Sampling was done every 30 min until the OOC of the reaction mixture reached more than 90% of the theoretical value, i.e., 5.47. In this study, the time taken for the epoxidation reaction to reach this point was 3 h. This method was adopted from Arniza et al., and Tuan Ismail et al., [10,21]. The epoxidised methyl oleate (EMO) was then separated from the spent acid and neutralised by washing with 1% sodium chloride solution and 0.5% sodium hydrogen carbonate solution until the pH of the EMO was about 6–7. Then, it was dried under vacuum for several hours at 80–90 °C. The white liquid product with a yield of 90 wt% was denoted as EMO. υ_max_/cm^−1^ 2924, 2855 (CH_2_), 1739 (C=O), 1463 (CH_2_), 1436 (CH_3_), 1170 (C-O), 827 (C-O-C), 723 (CH_2_); ^1^H-NMR (600 MHz, CDCl_3_): δ_H_ = 3.62 (3H, s, OCH_3_), 2.87–2.84 (2H, m, CH_2_CHOCHCH_2_), 2.26 (2H, t, O=CCH_2_CH_2_), 1.61–1.56 (2H, m, O=CCH_2_CH_2_CH_2_), 1.48–1.44 (4H, m, CHOCH_2_CH_2_), 1.31–1.21 (20H, m, CH_2_CH_2_CH_2_), 0.84 (3H, t, CH_2_CH_3_); ^13^C-NMR (150 MHz CDCl_3_): δ_C_ = 174.3 (CH_3_OC=O), 57.2–56.7 (CH_2_CHOCHCH_2_), 51.4 (CH_3_O), 34.1 (O=CCH_2_CH_2_), 31.9–29.0 (CH_2_CH_2_CH_2_), 27.8 (CH_2_CHOCHCH_2_), 24.9 (O=CCH_2_CH_2_CH_2_), 22.7 (CH_2_CH_3_), 14.1 (CH_2_CH_3_).

### 3.3. Ring-Opening of Epoxidised Methyl Oleate with Glycerol 

The reaction synthesis was carried out in a 1-L three-neck flask equipped with a magnetic stirrer and dropping funnel. An amount of 100 g of EMO was placed in the flask and preheated to a temperature of (90–120 °C) at a stirring speed of 200 rpm. A mixture of catalyst, Boron trifluoride diethyl ether complex (BF_3_·Et_2_O) (0.15–0.55%) and glycerol was added slowly to the preheated EMO. The mole ratio of the EMO to glycerol was (1:5–1:10). The reaction was started at the time when the mixture of glycerol and catalyst was added to the preheated EMO. The reaction mixture was sampled every hour to monitor the progress of reaction through the oxirane oxygen content (OOC) analysis. The reaction was considered completed when the OOC of the EMO was less than 0.1%. Then, the mixture component was poured into the separating funnel and left to separate into two layers. The bottom layer that contained excess glycerol was removed. The component was cooled to room temperature and ethyl acetate (100 mL) was added to dilute the component. Sodium chloride (200 mL) and sodium hydrogen carbonate (200 mL) was used in sequences to wash and neutralize the component until the pH was about 6–7. The component was dried over MgSO_4_. Ethyl acetate was removed by evaporation under vacuum to yield about 90 wt% as a yellowish liquid denoted as MOG-polyol. υ_max_/cm^−1^ 3391 (O-H), 2923, 2854 (CH_2_), 1740 (C=O), 1460 (CH_2_), 1436 (CH_3_), 1171 (C-O), 723 (CH_2_); ^1^H-NMR (600 MHz, CDCl_3_): δ_H_ = 3.81–3.78 (1H, m, CH_2_OCHOHCH_2_OH), 3.66–3.64 (1H, m, CHOHCH_2_OH), 3.59 (3H, s, OCH_3_), 3.48–3.44 (1H, m, CHOCH_2_CHOH), 3.43–3.40 (1H, m, CH_2_CHOHCHO), 3.06–3.03 (1H, m, CHOHCHOCH_2_)_,_ 2.23 (2H, t, O=CCH_2_CH_2_), 1.55–1.52 (2H, m, O=CCH_2_CH_2_CH_2_), 1.47–1.35 (2H, m, CHOCH_2_CH_2_), 1.31–1.18 (20H, m, CH_2_CH_2_CH_2_), 0.80 (3H, t, CH_2_CH_3_); ^13^C-NMR (150 MHz CDCl_3_): δ_C_ = 174.5 (CH_3_OC=O), 84.2 (CHOHCHOCH_2_), 73.5 (CH_2_CHOHCHO), 72.7 (CHOHCH_2_OH), 71.8 (CHOCH_2_CHOH), 71.1 (CH_2_OCHOHCH_2_OH), 51.5 (CH_3_O), 34.1 (O=CCH_2_CH_2_), 33.2 (CH_2_CH_2_CHOH), 31.9–29.0 (CH_2_CH_2_CH_2_), 24.8 (O=CCH_2_CH_2_CH_2_), 22.6 (CH_2_CH_3_), 14.1 (CH_2_CH_3_).

#### 3.3.1. Single Factor Experiment

Single factor experiments were performed to explore the effects of the following parameters on the hydroxyl value (OHV) response, namely mole ratio (EMO:Glycerol) (1:5, 1:7.5 and 1:10), catalyst (0.125%, 0.25%, 0.50%, 0.75% and 1.0%) and temperature (60 °C, 90 °C, 120 °C and 150 °C). A series of studies was then performed afterwards in order to find out the best reaction condition on hydroxyl value (OHV) using Response Surface Methodology (RSM). 

#### 3.3.2. Experimental Design and Optimisation via Response Surface Methodology (RSM)

Central composite rotatable design (CCRD) of RSM was employed to optimise the process parameter conditions of the three independent variables, namely mole ratio (EMO:Glycerol) (A), catalyst (B) and temperature (C). A summary of the independent variables and their coded level is illustrated in Table 8. This design was used to evaluate the combined effect of these variables towards the hydroxyl value (OHV) response (Y) of the MOG-polyol. A total number of 20 experiment runs (Table 9) based on CCRD was developed to identify the optimised levels of the significant independent variables and their relationship between independent variables generated by the Design-Expert version 7.0 Software (State Ease Inc., Minneapolis, MN, USA). These three independent variables were studied at three different levels. Therefore, the total 20 experiment runs involved 6 axial points, 6 central points and 8 factorial points, gained using Equation (3):

N = 2^n^ + 2n +n_c_ = 2^3^ + 2(3) + 6 = 20 (4)

where N is the total number of experiments run, n is number of factors and n_c_ is the number of replicates in the central points. The experiments were run randomly to minimise the influence of the extraneous factors [38].

#### 3.3.3. Statistical Analysis

Analysis of variance (ANOVA) was utilised to analyse the output data. By using ANOVA, it is a more reliable option to evaluate the quality of the fitted model. ANOVA is an important analysis in order to study the significance of the second order polynomial in the model. The second order polynomial regression model was used to fit the experimental data, and can be expressed using Equation (5):

(5) $$Y=\beta_{0}+\;\sum_{i=1}^{k}\beta_{i}x_{i}+\;\sum_{i=1}^{k}\beta_{ii}x_{i}^{2}+\;\sum_{i=1}^{k-1}\sum_{j>i}^{k}\beta_{ij}x_{i}x_{j}$$

where *Y* is the dependent variable (response); β_0_ is the constant coefficient; β_i_, β_ii_ and β_ij_ are coefficients of linear, quadratic and interaction terms, respectively; *k* is the number of tested variables (*k* = 4); *x_i_* and *x_j_* are the independent variables.

### 3.4. Verification of the Model

The verification of the final model was performed in order to inquire the adequacy of the predicted values. Three sets of the experiment data were suggested from the design software. The actual and predicted values were employed to calculate the percentage of residual standard error (RSE%), at less than 2%.

### 3.5. Characterisation

Wet chemistry analyses were performed following the American Oil Chemists’ Society Official (AOCS) methods [51]. The oxirane oxygen content (OOC) analysis of epoxidised methyl oleate (EMO) and methyl oleate-polyol (MOG-polyol) were determined according to AOCS Official method Cd 9-57. This analysis was conducted to measure the epoxide content of both samples. The iodine value (IV) was analysed following the AOCS Official method Cd 1d-92, in order to quantify the alkene group present in a sample and it was conducted on the MO and EMO. The acid value is defined as the amount of potassium hydroxide in milligrams needed to neutralise the free fatty acid in 1 g of sample. It was conducted according to the AOCS Official method Te 2a-64. The hydroxyl value (OHV) was determined following the AOCS Official method Cd 13-60, in order to quantify the hydroxyl group present in a sample. This analysis was performed on all the samples.

The infrared (FTIR) spectra of all samples, namely MO, EMO and MOG-polyol were investigated using a Perkin-Elmer FTIR Spectrum100 (Walthman, MA, USA) with an attenuated total reflectance (ATR) accessory. The FTIR was performed in the frequency range of 4000–650 cm^−1^ for 16 repeated scans with a resolution of 4 cm^−1^ in transmittance mode at room temperature. FTIR Spectroscopy was employed in order to identify the functional group that existed in the samples.

Nuclear magnetic resonance spectroscopy (^1^H-NMR) and (^13^C-NMR) was conducted using a JOEL JNM-ECZ600R (Peabody, MA, USA) at 600 MHz and 150 MHz, respectively. Deuterated chloroform was used as a solvent to dissolve the sample with approximately 10% *w*/*w* of sample. NMR Spectroscopy was utilized to identify the functional groups and the molecular structure of the samples. NMR analysis was performed on all samples, MO, EMO and MOG-polyol.

Gel permeation chromatography (GPC) was carried out using a Varian PL-GPC 50 Plus, (Polymer Laboratories Ltd., Church Stretton, Shropshire, UK) equipped with a differential refractive index (DRI)/viscometer and an autosampler combined with a detector. The sample was dissolved in THF with a concentration of 2 mg/mL. A PL gel mixed D column with THF as an eluent at a flow rate of 1 mL/minute was used to analyse the samples. The molecular weight was measured based on the universal calibration curve created using polystyrene standards with range from 162 to 1 × 10^5^ Da. Cirrus GPC/SEC system PL-GPC 50 plus (Shropshire, UK) was used to analyse the data. GPC was utilised to determine the molecular weight distribution and average molecular weight of a sample. This analysis was performed for all samples. 

The viscosity of a sample was measured by using a Brookfield Digital Rheometer, Model DV-111+ (Brookfield Engineering, Middleborough, MA, USA) at 25.0 °C ± 1. Approximately, 1 mL of sample was put on the sample platform for testing. A flat plate spindle (PP41) was moved onto the sample in rotation mode with a gap of 1 mm. The viscosity was measured using plate-plate geometry mode and analysed using Rheo Min Software (version 5.0, Middleborough, MA, USA). Viscosity analysis was performed on all samples. 

## 4. Conclusions

The optimum reaction conditions for the synthesis of bio-polyol (MOG-polyol) by applying Response surface methodology (RSM) with a combination of central composite rotatable design (CCRD) with three variables at five levels (−2, −1, 0, +1, +2) was successfully carried out. The effect of the three main variables was investigated using an empirical model and it showed a linear effect on the response (hydroxyl value). Based on the ANOVA analysis, the predicted model was fitted, with the *p*-value > 0.0001 and *F*-value (106.47), and an insignificant lack of fit and a high coefficient of determination (R^2^) = 0.9897. The verification of this model was achieved by testing three experiments suggested by RSM, and the RSE obtained was less than 2%. The optimised reaction condition for synthesis MOG-polyol was 1:10 (mole ratio of EMO to glycerol), 0.18% of catalyst and 120 °C (temperature) which resulted a 306.19 mg KOH/g of hydroxyl value (OHV) and a desirability of 0.976. The molecular structure of the MOG-polyol was confirmed by FTIR and NMR analysis. Therefore, from this study, the result obtained showed that RSM is an excellent tool to optimise the reaction conditions for synthesising the bio-polyol (MOG-polyol) from methyl oleate as the model provided a desirable prediction of the hydroxyl value (OHV). Hence, a scaled-up process for MOG-polyol may be carried out using these optimum reaction conditions. 

## Figures and Tables

**Table 1:** Experimental data of actual and predicted values of hydroxyl value of MOG-polyol.

	Independent Variable			Hydroxyl Value (mg KOH/g)	
Run Order	Mole Ratio (EMO:Glycerol)	Catalyst (%)	Temperature (°C)	Actual	Predicted
1	1:3.30	0.35	120.00	254.95	252.90
2	1:7.50	0.35	120.00	282.40	280.83
3	1:5.00	0.55	150.00	232.02	234.87
4	1:7.50	0.35	120.00	281.82	280.83
5	1:5.00	0.15	90.00	272.51	274.68
6	1:7.50	0.35	69.50	272.27	271.21
7	1:10.00	0.15	150.00	299.00	297.66
8	1:10.00	0.15	90.00	303.53	299.92
9	1:7.50	0.35	120.00	282.40	280.83
10	1:7.50	0.35	120.00	278.35	280.83
11	1:7.50	0.69	120.00	233.72	231.84
12	1:7.50	0.35	120.00	281.82	280.83
13	1:7.50	0.35	120.00	278.35	280.83
14	1:11.70	0.35	120.00	305.04	308.15
15	1:10.00	0.55	90.00	278.83	280.85
16	1:7.50	0.01	120.00	289.95	292.89
17	1:10.00	0.55	150.00	278.26	275.34
18	1:5.00	0.55	90.00	223.80	224.39
19	1:5.00	0.15	150.00	291.19	288.41
20	1:7.50	0.35	170.50	275.99	278.12

**Table 2:** ANOVA results for quadratic model developed for Hydroxyl value of MOG-polyol.

Source	Sum of Squares	df	Mean Square	*F*-Value	*p*-Value	Significant
Model	9517.62	9	1057.51	106.47	<0.0001	*
A	3685.25	1	3685.25	371.04	<0.0001	*
B	4499.43	1	4499.43	453.02	<0.0001	*
C	57.64	1	57.64	5.8	0.0367	**
AB	487.34	1	487.34	49.07	<0.0001	*
AC	128	1	128	12.89	0.0049	*
BC	5.28	1	5.28	0.53	0.4826	***
A^2^	0.16	1	0.16	0.016	0.9014	***
B^2^	613.76	1	613.76	61.79	<0.0001	*
C^2^	68.43	1	68.43	6.89	0.0254	**
Residual	99.32	10	9.93			
Lack of Fit	80.14	5	16.03	4.18	0.0714	***
Pure Error	19.19	5	3.84			
Corrected Total	9616.94	19				

Note: *** in the table indicates insignificant difference at *p*-value > 0.05; ** indicates significant difference at 0.05 level; * indicates significant difference at 0.01 level.

**Table 3:** Regression coefficients of final reduced model.

**SD**	3.15	**R^2^**	0.9897
**Mean**	274.81	**Adjusted R^2^**	0.9804
**CV%**	1.15	**Predicted R^2^**	0.9296
**PRESS**	677.12	**Adequate Precision**	37.588

**Table 4:** Constraints applied for optimisation.

Independent Variables	Goal	Lower Limit	Upper Limit
Mole ratio (A)	In range	5.00	10.00
Catalyst (B)	In range	0.15	0.55
Temperature (C)	In range	90.00	150.00
Hydroxyl value	Maximise	223.80	305.04

**Table 5:** Actual and predicted responses at optimum combinations.

Exp	Independent Variable			Hydroxyl Value (mg KOH/g)		RSE (%)
	A	B (%)	C (°C)	Actual	Predicted	
1	1:10.00	0.18	120	306.19	301.248	1.64
2	1:10.00	0.19	120	304.06	301.246	0.93
3	1:10.00	0.20	120	303.03	301.225	0.60

Note: In the table indicates A: Mole ratio (EMO:Glycerol); B: Catalyst; C: Temperature.

**Table 6:** Optimum condition for synthesis of MOG-polyol.

Independent Variable			Hydroxyl Value (mg KOH/g)		Desirability
A	B (%)	C (°C)	Actual	Predicted	
1:10.00	0.18	120	306.19	301.248	0.976

Note: In the table indicates A: Mole ratio (EMO:Glycerol); B: Catalyst; C: Temperature.

**Table 7:** Characteristics of MO, EMO and MOG-polyol.

Parameter	MO	EMO	MOG-Polyol
Iodine value (g I_2_ 100/g)	91.86	0.63	nd
Oxirane oxygen content (%)	nd	5.10	0.05
Acid value (mg KOH/g)	1.12	1.16	1.18
Hydroxyl value (mg KOH/g)	16.50	20.60	306.19
Viscosity at 25 °C (m Pa)	5.49	13.59	513.48
Average molecular weight, MW (Da)	315	297	488
Polydispersity Index	1.0096	1.0175	1.1402

Note: nd in the table indicates ‘not done’.

**Table 8:** Summary of independent variables and their coded levels.

Independent Variable	Units	Coded Level				
		−2	−1	0	+1	+2
A	-	3.30	5.00	7.50	10.00	11.70
B	%	0.01	0.15	0.35	0.55	0.69
C	°C	69.50	90.00	120.00	150.00	170.50

Note: In the table indicates A: Mole ratio (EMO:Glycerol); B: Catalyst; C: Temperature.

**Table 9:** CCRD design matrix with 20 experiments.

	Independent Variable		
Run Order	Mole Ratio (EMO:Glycerol)	Catalyst (%)	Temperature (°C)
1	1:3.30	0.35	120.00
2	1:7.50	0.35	120.00
3	1:5.00	0.55	150.00
4	1:7.50	0.35	120.00
5	1:5.00	0.15	90.00
6	1:7.50	0.35	69.50
7	1:10.00	0.15	150.00
8	1:10.00	0.15	90.00
9	1:7.50	0.35	120.00
10	1:7.50	0.35	120.00
11	1:7.50	0.69	120.00
12	1:7.50	0.35	120.00
13	1:7.50	0.35	120.00
14	1:11.70	0.35	120.00
15	1:10.00	0.55	90.00
16	1:7.50	0.01	120.00
17	1:10.00	0.55	150.00
18	1:5.00	0.55	90.00
19	1:5.00	0.15	150.00
20	1:7.50	0.35	170.50

## Data Availability

Not applicable for this study.
